# Improving influenza forecast in the tropics and subtropics: a case study of Hong Kong

**DOI:** 10.1098/rsif.2024.0649

**Published:** 2025-01-15

**Authors:** Haokun Yuan, Eric H. Y. Lau, Benjamin J. Cowling, Wan Yang

**Affiliations:** ^1^ Department of Epidemiology, Mailman School of Public Health, Columbia University, New York, NY, USA; ^2^ WHO Collaborating Centre for Infectious Disease Epidemiology and Control, School of Public Health, Li Ka Shing Faculty of Medicine, The University of Hong Kong, Hong Kong Special Administrative Region, People’s Republic of China; ^3^ Laboratory of Data Discovery for Health Limited, Hong Kong Science Park, New Territories, Hong Kong Special Administrative Region, People’s Republic of China; ^4^ School of Health & Social Development, Deakin University, Melbourne, Victoria, Australia

**Keywords:** influenza, long-range forecast, seasonality, climate, humidity, temperature

## Abstract

Influenza forecasts could aid public health response as shown for temperate regions, but such efforts are more challenging in the tropics and subtropics due to more irregular influenza activities. Here, we built six forecast approaches for influenza in the (sub)tropics, with six model forms designed to model seasonal infection risk (i.e. seasonality) based on the dependence of virus survival on climate conditions and to flexibly account for immunity waning. We ran the models jointly with the ensemble adjustment Kalman filter to generate retrospective forecasts of influenza incidence in subtropical Hong Kong from January 1999 to December 2019 including the 2009 A(H1N1)pdm09 pandemic. In addition to short-term targets (one to four weeks ahead predictions), we also tested mid-range (one to three months) and long-range (four to six months) forecasts, which could be valuable for long-term planning. The largest improvement came from the inclusion of climate-modulated seasonality modelling, particularly for the mid- and long-range forecasts. The best-performing approach included a seasonal-trend-based climate modulation and assumed mixed immunity waning; the forecast accuracies, including peak week and intensity, were comparable to that reported for temperate regions including the USA. These findings demonstrate that incorporating mechanisms of climate modulation on influenza transmission can substantially improve forecast performance in the (sub)tropics.

## 1. Introduction

To support public health response and mitigate the disease burden associated with annual influenza epidemics, influenza forecasts have been increasingly used since the 2010s in places such as the USA [1,2]. Such forecasts often include influenza incidence and/or hospitalizations in future weeks (i.e. *n*-week ahead predictions) and seasonal targets such as epidemic onset week, peak week and peak intensity [2]. Forecasters often use a model to capture epidemic dynamics based on available observations up to the time of forecast initiation (i.e. model calibration or training) and then use the calibrated model to predict future epidemic trajectories (typically, one to four weeks ahead predictions, as in the US forecasts hosted by Centers of Disease Control and Prevention [3,4]). Thus, an improved model—e.g. through more accurate modelling of influenza seasonality—could improve influenza forecast accuracy. For instance, Shaman *et al*. developed a humidity-forced epidemic model [5,6] to describe how humidity modulates influenza transmission in temperate regions based on laboratory studies [7–9], and showed this model is able to more accurately characterize influenza epidemics and improve forecasts in the USA [5,6,10] and other temperate countries [11].

Influenza seasonality is less well-characterized in tropical regions [12] and influenza virus could circulate most of the year in subtropical regions [13], making forecasts more challenging for (sub)tropical regions [11,14]. To better understand influenza seasonality in the (sub)tropics, previous studies [15–19] have reported a ‘U-shaped’ dependence of influenza transmission on humidity (i.e. higher transmission at both very low and very high humidity conditions), consistent with the bimodal epidemic pattern observed. In a 2021 study [20], we developed a climate-modulation model to capture this ‘U-shaped’ dependence on humidity [15–18] and the additional impact of temperature (i.e. better viral survival at lower temperatures [7,21]), and showed the model is able to capture long-term influenza dynamics observed over 20 years in subtropical Hong Kong. In addition, we also developed a simple model to more flexibly account for short-term (cross)-immunity in this prior work [20].

In this study, we build on the [20] models to develop forecast approaches incorporating climate conditions and different immunity-waning assumptions. We test whether the inclusion of these model components can improve influenza forecasting for the (sub)tropics by generating retrospective forecasts of influenza incidence in Hong Kong from January 1999 to December 2019 including the 2009 A(H1N1)pdm09 pandemic. Importantly, we note several distinctions of this study from our previous work in [20] and other forecasts. There are additional challenges facing forecast. The first is to estimate the initial conditions (e.g. population susceptibility and virus transmissibility) at forecast initiation to capture the real-time epidemic dynamics (versus fit to all historical data in [20]). Another challenge is to account for forecast uncertainties such as changes in virus transmissibility over time due to changes in circulating influenza strains (versus average estimate over multiple years per historical data in [20]). Here, we address these challenges using the ensemble adjustment Kalman filter (EAKF) [22], a data assimilation method that affords system state estimation based on incidence data as well as ensemble forecast to account for forecast uncertainty (see §2). In addition, unlike most current forecasts focusing on short-term targets (e.g. one to four weeks ahead [3,4]), we generate forecasts for 1–26 weeks (i.e. up to six months) in the future, to additionally test mid-range (e.g. one to three months) and long-range (e.g. four to six months) forecasts, which could be valuable for long-term planning.

## 2. Methods

### 2.1. Data

Sentinel influenza surveillance data (from the first week of 1998 to the last week of 2019) were collected by the Centre for Health Protection in Hong Kong. These included the weekly proportion of outpatients presenting with influenza-like illness (ILI; defined as fever greater than 38.0°C plus cough and/or sore throat) and the proportion of respiratory samples testing positive for influenza viruses [23–25]. We multiplied weekly ILI consultation rates and viral detection rates to obtain a more specific measure, ILI+, following previous studies [26,27]. To account for changes in detection efforts during the 2009 A(H1N1)pdm09 pandemic, we adjusted ILI+ during June to November 2009 using estimated attack rate over the same period from Wu *et al*. [28].

Daily temperature and relative humidity data were sourced from the Hong Kong Observatory [29]. We computed specific humidity using temperature and relative humidity per the Clausius–Clapeyron equation [30]. We further averaged the data over the 21 years study period for each day of the year and used these climatological daily temperature and specific humidity in the climate-modulation models (see §2.2. below). Note that we used climatological values, because real-time measures are not available for the extended forecast period (up to six months here) during real-time forecasts and that previous work has demonstrated the superior performance of forecast approaches using climatological compared with real-time humidity [10]. In addition, we used instantaneous temperature and humidity with no lag time, because we directly modelled the survival of influenza virus and transmission rate under the corresponding climate conditions.

### 2.2. Model-filter forecast approaches (model training)

We developed six model-filter forecast approaches using six epidemic models (three climate-modulation settings × two immunity-related settings) and the EAKF [22] to train the model before generating a forecast (see electronic supplementary material, table S1 for a comparison of the model settings and parameters estimated by the EAKF). The base epidemic model assumed the susceptible-infectious-recovered-susceptible (SIRS) construct [20],


(2.1) $$\left\{ \begin{array}{l} \frac{dS}{dt}=\frac{N-S-I}{L}-\frac{R_{0}\left(t\right)}{D}\frac{I^{p}S}{N}-\alpha +\mu \left(N-S\right)\\ \frac{dI}{dt}=\frac{R_{0}\left(t\right)}{D}\frac{I^{p}S}{N}-\frac{I}{D}+\alpha -\mu I, \end{array}\right.$$

where *S*, *I* and *N* are the number of susceptible, infectious and total population, respectively; t is time in days. *L* and *D* are the immunity and infectious period, respectively. $R_{0}\left(t\right)$ is the basic reproduction number (i.e. the average number of secondary infections per primary infection in a naive population) at time *t*; $R_{0}\left(t\right)$ could vary over time depending on circulating strains (e.g. A/H3N2 may be more transmissible than A/H1N1 [31,32]) and climate conditions (see e.g. equation 2.2). Relatedly, $R_{0}/D$ is the transmission rate at time *t*. The exponent p (set to 0.97, as done in [20]) is used to account for imperfect population mixing [33]; α is a random seeding from outside population (i.e. travel-related importation of infection, nominally set to 1 per 10 days as in [20]; note, α has little impact on the epidemic dynamics, as shown in [20]); and μ is the birth and death rate. Using the model, we simulated influenza incidence (per 100 000 population) and converted it to simulated ILI+ to match the observed ILI+, as done in [26,27]; here, we estimated the conversion factor using the EAKF (see below).

We tested three climate modulations. The first, baseline setting (referred to as the ‘null’ model) included no climate variables and allowed $R_{0}$ to vary over time as estimated by the EAKF based on ILI+ data. The second setting used a humidity- and temperature-forced (AH/T) function developed in [20] per

(2.2) $$R_{0}\left(t\right)=\left[aq^{2}\left(t\right)+bq\left(t\right)+c\right]{\left[\frac{T_{c}}{T\left(t\right)}\right]}^{T_{exp}}.$$

Here, the parabola in the first set of brackets describes the ‘U-shaped’ relationship of influenza transmission (represented by $R_{0}$) with humidity [15–19] (*q* is the ambient specific humidity)*.* We reparametrized the coefficients *a*, *b*, and *c* in equation (2.2) using the maximal $R_{0}\;(R_{0max})$ and the difference between the maximal and minimal $R_{0}\;(R_{0diff})$ [20], and estimated $R_{0max}$ and $R_{0diff}$ using the EAKF. The second set of brackets in equation (2.2) describes the impact of temperature (*T*; assuming temperature lower than a threshold $T_{c}$ would facilitate influenza transmission and otherwise inhibit transmission; the exponent $T_{exp}$ controls the strength of this modulation) [20]. For the third setting, we scaled the weekly $R_{0}(t)$ estimates (using equation (2.2), parameters estimated in [20], and climatological humidity and temperature data) by dividing the yearly mean to obtain a seasonal trend $s_{t}=R_{0}(t)/\bar{R_{0}(t)}$; we then incorporated this seasonal trend in the epidemic model by replacing $R_{0}(t)$ in equation (2.1) with $R_{0,\;strain}(t)s_{t}$. In doing so, we decoupled $R_{0}$ into two components—i.e. $R_{0,\;strain}$ is the strain-related component and was estimated along with other parameters using the EAKF, and $s_{t}$ is the pre-estimated seasonal-trend-based climate modulation. We refer to this model as the $\tilde{AH/T}$ model.

We included two model forms related to immunity [20]. The first assumed immunity wanes exponentially per the term N−S−I/L (i.e. using equation (2.1)). The second model (referred to as ‘Vary’; table 1) assumed a portion (*ρ*) of recoverees lose immunity after a short period (*L_s_*) of full protection and the rest lose immunity exponentially. Accordingly, the second model replaces N−S−I/L in equation (2.1) with the term

(2.3) $$\rho \frac{\beta_{t-L_{s}}I_{t-L_{s}}^{p}S_{t-L_{s}}}{N}+\left(1-\rho \right)\frac{N-S-I}{L}$$

**Table 1.** Performance of the seven forecast approaches, and the best setting selected for each forecast approach. The rankings are not necessarily integers, because they are average over the corresponding individual targets. OEV: observation error variance.

model	main model components	overall	accuracy		log score		WIS		settings		
			seasonal	weekly	seasonal	weekly	seasonal	weekly	OEV (B; M)	λ	γ
$SIRS\left(\tilde{AH/T}/Vary\right)$	seasonal trend based on humidity and temperature; mixed immunity waning	1.7	2.3	2.1	1.7	1.2	1.5	1.2	30 000; 20	1.01	1
$SIRS\left(\tilde{AH/T}\right)$	seasonal trend based on humidity and temperature; exponential immunity waning	2.5	2.2	2.8	2.0	3.4	2.5	2.2	1000; 20	1.05	1
SIRS(AH/T)	humidity and temperature modulation; exponential immunity waning	3.4	3.2	3.2	4.3	2.5	3.5	3.4	1000; 20	1.03	0.95
SIRS(AH/T/Vary)	humidity and temperature modulation; mixed immunity waning	3.7	4.0	4.0	4.7	2.8	3.7	3.2	100; 20	1.05	0.9
SARIMA^a^	benchmark time series model (with seasonal trend)	4.8	4.0	3.5	5.0	5.4	5.2	6.0			
SIRS(null/Vary)	no climate modulation; mixed immunity waning	5.7	6.0	6.2	5.3	5.8	5.8	5.3	10 000; 30	1.04	1
SIRS(null)	no climate modulation; exponential immunity waning	6.2	6.3	6.3	5.0	6.8	5.8	6.7	30 000; 30	1.02	1

^a^ The SARIMA model includes different parameters and was optimized differently (see details in the main text).

where $\beta_{t-L_{s}}I_{t-L_{s}}^{p}S_{t-L_{s}}$ represents the number of people who were infected *L_s_* days ago and lost their immunity on day *t* (i.e. after an immunity period of *L_s_* days).

We ran each SIRS model jointly with the EAKF [22] to estimate model state variables (i.e. *S* and *I*, and incidence in equation (2.1)) and parameters (e.g. *R*_0_) for each week. Briefly, we first initiated the SIRS-EAKF system with an ensemble of the model realizations (*n* = 500 ensemble members here) using variables and parameters randomly drawn from the prior ranges specified in electronic supplementary material, table S1 for the first week of 1998. Each week, we integrated the SIRS model forward by one week to generate the model-predicted state (i.e. the prior) and updated the system (all state variables and parameters) using the prior and the observation for that week (i.e. the likelihood) per Bayes’ rule [22]. We repeated this filtering process to assimilate new observations each week, continuously from the first observation (here, at the first week of 1998) to the week of forecast initiation.

### 2.3. Retrospective forecast generation

After assimilating the last available observation, we halted the filtering process and integrated the epidemic model ensemble forward for 26 weeks (i.e. six months; see additional processing below) with the last model state estimates to generate a forecast. Each forecast included the median (i.e. point prediction) and probability distribution (i.e. probabilistic forecast) of weekly ILI+ for the 26 weeks (i.e. 1–26 weeks ahead prediction) and of the seasonal targets (i.e. ILI+ peak week, peak intensity and cumulative total) for the next three months and next six months, separately. We computed the median prediction and constructed the probability distributions directly using the model ensemble. In total, we generated 1148 forecasts, initiated every week from the first week of 1999 (i.e. after a minimum of 1 year training period) through the 26th week of 2019 (i.e. the last six months forecast extended to the end of 2019). To account for stochasticity from the system initiation and model simulation (all models were run stochastically), we repeated each forecast 10 times.

### 2.4. Testing of the model-filter forecast approaches

To optimize each SIRS-EAKF forecast approach, we tested three main factors affecting the EAKF performance and distribution of the forecast ensemble. First, in the EAKF algorithm, the observation error variance (OEV), often set heuristically due to a lack of data, partly determines the posterior distribution of model state variables and parameters. Here, we computed the OEV as ${\text{OEV}}_{t}=B+M\times \sum_{j=t-1}^{t-3}\frac{ILI_{+j}}{3}$, i.e. a baseline error (*B*) plus *M* times the mean ILI+ during the preceding three weeks, and tested 18 combinations of *B* and *M* (*B* = 100, 1000, $1\times 10^{4}$, $2\times 10^{4}$, $3\times 10^{4}$, or $4\times 10^{4}$; *M* = 10, 20 or 30). Second, to improve EAKF performance, we applied covariance inflation [34,35]—i.e. increased the covariance of model ensemble by a factor *λ* (>1), which affects the posterior distribution and in turn forecast distribution. To examine this impact, we tested eight covariance inflation settings, including using adaptive covariance inflation (i.e. λ is estimated by the filter) [35,36], and seven fixed inflation factors (λ = 1.01, 1.02, …, or 1.07). Finally, as done in [37], we applied a deflation technique—i.e. reduced the spread of the state variables by a factor γ (<1) during each week of the forecast period. The deflation method was initially proposed to counter long-term error growth and tested for long-range COVID-19 forecast [37]. Here, similar challenges could apply to long-range influenza forecast. In addition, by design, deflation would affect the forecast distribution. Thus, here we tested three related settings (i.e. γ = 1, 0.95 or 0.9). In total, we tested 432 settings (i.e. 18 OEV settings × eight λ values × three γ values) for each SIRS-EAKF forecast approach.

### 2.5. Forecasting using a seasonal autoregressive integrated moving average model

To benchmark the forecast performance, we also generated forecasts using a seasonal autoregressive integrated moving average (SARIMA) model. We included a seasonal cycle of 52 weeks to account for influenza seasonality and optimized the SARIMA model using the auto.arima function in the ‘forecast’ R package [38,39]. To generate each forecast, we simulated the model forward in time for 26 weeks with 10 000 bootstrapping runs (versus only 500 runs in the EAKF-based forecasts) to account for forecast uncertainty.

### 2.6. Forecast calibration

To ensure the reliability of a forecast, forecast probability should align with the observed event frequency (i.e. a forecast that an event will occur with a *x*% probability should cover the event approximately *x*% of the time). A typical examination is to plot the forecast probability against the observed event frequency (i.e. the calibration curve) and check if this curve is close to the 1 : 1 diagonal line. To do so, we segregated the *n*-week ahead forecasts by prediction interval (PI: 10%, 20% … 90%, 95% and 98%) and computed the frequency of observations falling within each of these PIs (i.e. the actual coverage). Given the large number of forecast approaches and settings tested, we used the relative root mean square error (rRMSE) between the PIs and the actual coverage to screen those that would have the calibration curve close to the 1 : 1 diagonal line; only those with an rRMSE less than 0.2 were included in further forecast comparison.

### 2.7. Forecast evaluation

To evaluate forecast performance, we computed the point prediction accuracy [10,37], probabilistic logarithm score (log score) [2] and weighted interval score (WIS) [40] for each forecast target (i.e. 26 *n*-week ahead weekly targets and six seasonal targets). To compute the point prediction accuracy, we tallied the percentage of forecasts falling within a preset tolerance. In general, a forecast was considered accurate for peak timing if the predicted value was within ±1 week of the observed, and for other targets (i.e. weekly incidence, peak intensity or the cumulative total) if the predicted was within ±25% of the observed. However, as the forecasts were generated throughout the year, some could cover a non-epidemic period (defined as ILI+ less than 500 cases per 100 000 people for all weeks) with no meaningful epidemic peak. For such periods, a forecast was considered accurate for the peak timing and peak intensity if there was no predicted epidemic (i.e. predicted ILI+ less than 500 cases per 100 000 people for all weeks). Similarly, for weeks with low ILI+ (defined as ILI+ less than 100 cases per 100 000 people), a weekly ILI+ forecast was considered accurate if the predicted value was less than 125 cases per 100 000 people (i.e. 1.25 times of the preset lower bound). To compute the log score, we binned the forecast ensemble to generate the probability distribution and took the logarithm of the probability density of the bins capturing the observation (electronic supplementary material, table S2). The WIS measures the distance between the forecast distribution and the observation, i.e. akin to the absolute error of a point prediction [40]. For the WIS, we used equation 1 in [40] and included 11 intervals (i.e. 10%, 20% … 90%, 95% and 98%). We constructed the 95% confidence interval for each evaluation metric (accuracy, log score or WIS), using 100 random samples drawn from all forecasts for the corresponding target. Compared with using repeated runs, such bootstrap estimates additionally account for the variation in performance during different epidemic phases (e.g. forecasts around the peak week versus during weeks with low influenza activity).

As noted above, influenza epidemics could occur year-round in subtropical regions such as Hong Kong. To examine performance differences by season, we also segregated the forecasts into two approximately defined ‘seasons’ based on temperature and ILI+ data (i.e. events occurring during weeks 21–46—approximately from mid-May to mid-November when temperature tends to be higher and influenza incidence tends to be lower—were classified as ‘warm season’ and otherwise ‘cool season’) and computed the above three evaluation metrics by season.

### 2.8. Comparing the forecast approaches

To determine the best setting (from 432 combinations tested) for each SIRS-EAKF forecast approach, we first used each metric (accuracy, log score or WIS) to rank the settings for each target, averaged the target-specific rankings by target type (i.e. seasonal and weekly targets separately) and further averaged the two target-type-specific rankings to obtain a final metric-level ranking (note this scheme essentially placed less weight on each weekly target, given the larger number of weekly targets in this study). We then averaged the three metric-level rankings and selected the setting with the highest overall ranking to use for each forecast approach.

We ranked the seven forecast approaches (six SIRS-EAKF approaches, each per their best setting; and the optimized SARIMA model), using the same ranking procedure above. Additionally, we performed a Friedman test followed by a Nemenyi test to assess whether forecast performance (based on each metric) differed significantly between each pair of forecast approaches [10]. We performed this analysis by target type using each evaluation metric, and overall combining all forecast targets using accuracy and log score. Since WIS (equivalent to absolute errors) for different forecast targets are on different scales, for the WIS-based overall statistical tests, we combined *n*-week ahead forecasts alone.

## 3. Results

### 3.1. Example forecasts and overall forecast performance

**Figure 1.**
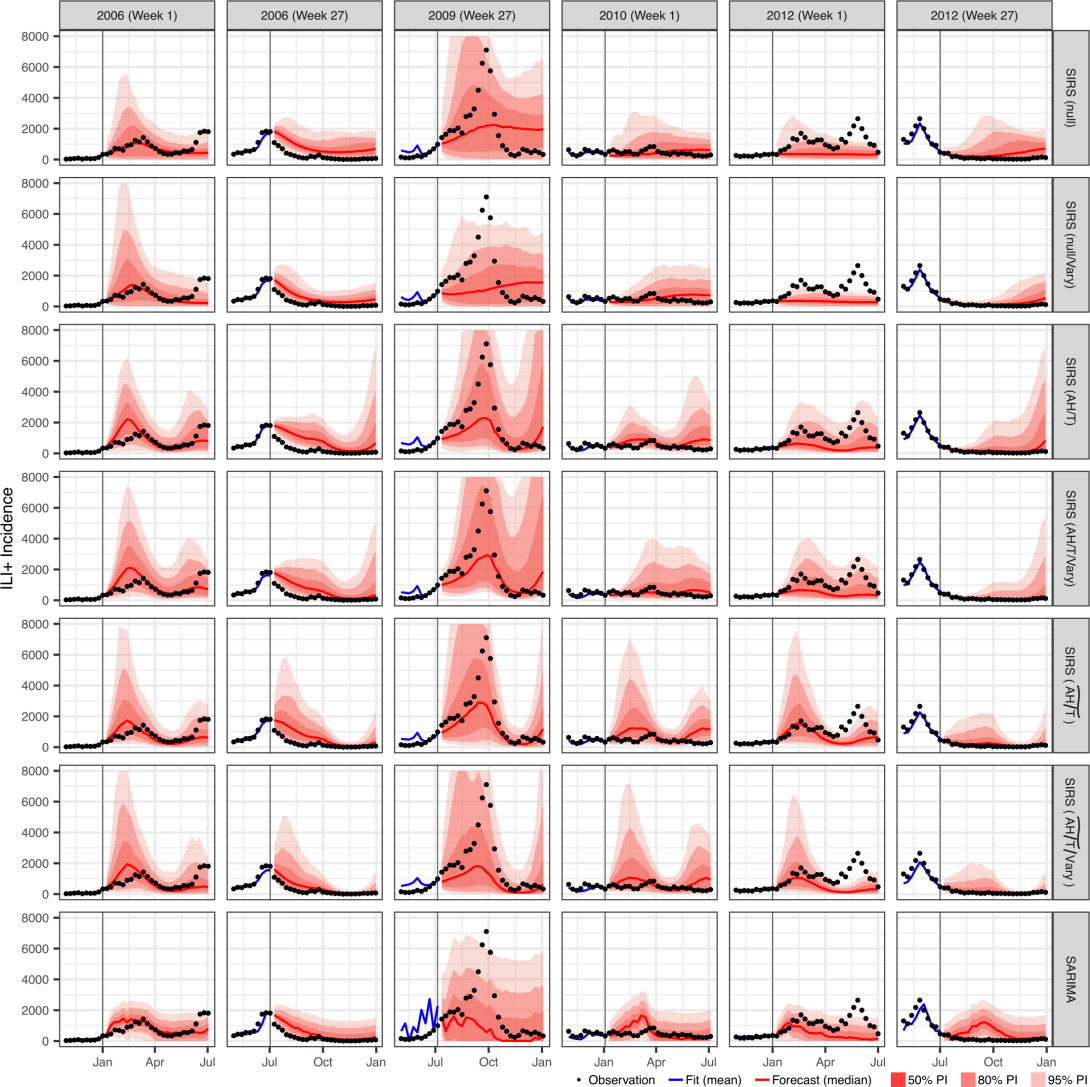
Example forecasts. Each panel shows a forecast (1–26 weeks ahead) generated by an approach (see the name of the approach used on the right) at a given time point (see specific timing on the top). Vertical lines indicate the week of forecast initiation. Dots show reported weekly influenza incidence (ILI+), including for the forecast period. Blue lines show mean model training estimates. Red lines and red areas show model forecasts: line = median; red areas (darker to lighter) = 50%, 80% and 95% predictive interval (PI).

Influenza epidemics are highly diverse in Hong Kong (see weekly ILI+ during the entire study period in electronic supplementary material, figure S1). As examples, figure 1 shows forecasts generated at two time points (at first and 27th week of the year) for three periods, compared with the observed ILI+. These included two epidemics in year 2006 (figure 1, first two columns), the 2009 A(H1N1)pdm09 pandemic (middle two columns) and a large bimodal epidemic after the 2009 pandemic (last two columns). Given the diverse dynamics, the forecast approaches (six SIRS-EAKF approaches and one using a SARIMA model) had varying success predicting the short- and long-term epidemic trajectories, depending on the epidemics and timing of forecast.

**Figure 2.**
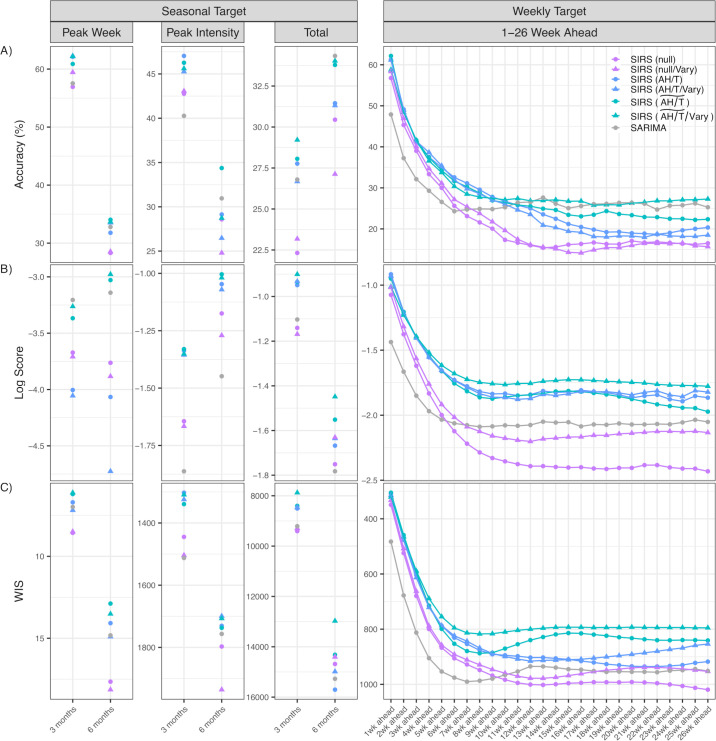
Forecast performance for each target, aggregated over all forecasts by each approach, evaluated using (*a*) accuracy, (*b*) log score and (*c*) WIS. Left panel shows forecast performance for the seasonal targets (i.e. peak week, peak intensity and cumulative total; see names on the top) for two forecast time windows (i.e. three or six months; see *x*-axis). Right panel shows forecast performance for the weekly targets (1–26 weeks ahead forecasts; see *x*-axis). Colours and shapes of the dots denote forecast approaches (see legend). Note that here accuracy and log score are positively oriented (i.e. higher values indicate better performance) whereas WIS is negatively oriented (i.e. smaller values indicate better performance). For ease of comparison, we reverse the order of the *y*-axis for WIS in (*c*) such that, in all subplots, approaches with superior performance are displayed on top.

Table 1 shows the best setting selected for each approach and performance ranking, tallying all forecasts generated during the 21 years study period (i.e. January 1999–December 2019). Calibration curves (based on all *n*-week ahead predictions) indicate that all seven forecast approaches can align the prediction intervals with the observed event frequencies (electronic supplementary material, figure S2). Figure 2 shows forecast accuracy, log score and WIS for each target, aggregating all forecasts generated by each approach. Performances were similar when segregated by season, despite different influenza activities during winter and summer (electronic supplementary material, figure S3), and consistent across epidemic phases as indicated by the tight confidence intervals (electronic supplementary material, figure S4). Finally, the parameter estimates also are in line with values reported in the literature for influenza (e.g. 2–4 days of generation time or infectious period [41]; see posterior estimates for parameters in the best-performing approach in electronic supplementary material, figure S5).

### 3.2. Factors affecting influenza forecast performance

**Figure 3.**
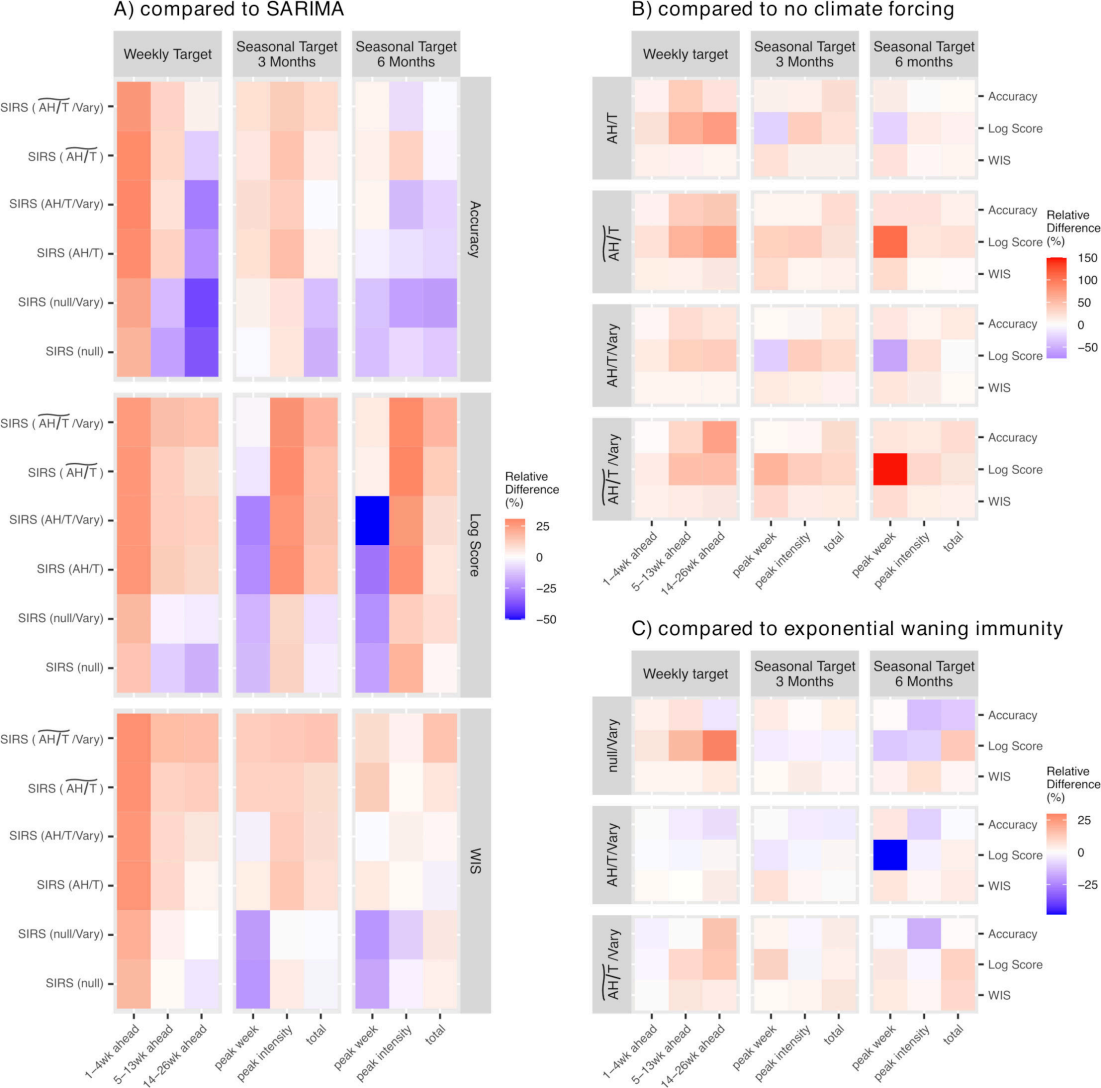
Factors affecting influenza forecast performance: (*a*) model type, comparing six SIRS-EAKF approaches (see names in left *y*-axis labels) and the SARIMA approach; (*b*) inclusion of seasonality, comparing SIRS-EAKF approaches with a climate modulation (see seasonality/immunity setting in left *y*-axis labels) versus without that climate modulation; (*c*) model assumptions on waning immunity, comparing SIRS-EAKF approaches assuming varying immunity-waning mechanisms (see seasonality/immunity setting in left *y*-axis labels) versus exponential waning. Heat maps show the per cent difference in forecast accuracy, log score and WIS (see specific metric used in right *y*-axis labels) between two comparison forecast approaches. Positive percentages (red colours) indicate a given approach outperforms its comparison approach.

We next examine how model types and structures could affect influenza forecast performance. First, all SIRS-EAKF approaches outperformed the benchmark SARIMA approach in predicting short-term targets (i.e. the one to four weeks ahead predictions; table 2 and figure 2) and mid-range targets (i.e. weekly predictions up to three months ahead and most three months seasonal targets; figure 3*a*), suggesting mechanistic epidemic models (e.g. SIRS models) are able to better capture recent influenza transmission dynamics than statistical (e.g. ARIMA) models. Second, inclusion of climate conditions to model influenza seasonality substantially improved forecast performance (figure 3*b* and electronic supplementary material, table S3). The improvement was evident for both climate-forced seasonality models (AH/T and $\tilde{AH/T}$), and more substantial for $\tilde{AH/T}$ (overall, 23% higher accuracy, 51% higher log score, 15% lower WIS versus the SIRS (null) model; electronic supplementary material, table S3). In addition, the climate modulations also substantially improved the long-range forecasts (e.g. 14–26 weeks ahead), allowing the climate-forced SIRS models to also outperform the SARIMA model in predicting long-range targets, particularly when evaluated per log score and WIS (figure 3*a*, bottom two panels, more red cells in the first four rows of each panel). Third, inclusion of mixed immunity assumptions also appeared to help improve forecast performance (figure 3*c*, more red cells); however, the improvements were not substantial (electronic supplementary material, table S3).

**Table 2.** Comparison of forecast performance by the best-performing SIRS ($\tilde{AH/T}/Vary$)-EAKF approach and two base approaches (i.e. SARIMA and SIRS (null)-EAKF). Numbers show the mean point prediction accuracy, log score or WIS (see column ‘metric’) of forecasts generated using the SIRS ($\tilde{AH/T}/Vary$)-EAKF approach versus the comparison approach (see column title), and the per cent difference of the two quantities in the parentheses (a positive percentage indicates the SIRS ($\tilde{AH/T}/Vary$)-EAKF approach performs better); asterisks indicate if the difference is significant at α = 0.05 (*), 0.01 (**) or 0.001 (***) level, using a Friedman test followed by a Nemenyi test.

target	metric	$SIRS\left(\tilde{AH/T}/Vary\right)$ versus SARIMA	$SIRS\left(\tilde{AH/T}/Vary\right)$ versus SIRS(null)
overall	accuracy	0.33 versus 0.32 (3.09%)***	0.33 versus 0.27 (23.66%)***
	log score	−2.19 versus −2.51 (38.79%)***	−2.19 versus −2.68 (63.69%)***
	WIS	746 versus 911 (18.14%)***	746 versus 918 (18.7%)***
1–4 weeks ahead	accuracy	0.46 versus 0.37 (26.59%)***	0.46 versus 0.44 (6.32%)***
	log score	−1.75 versus −2.23 (63.02%)***	−1.75 versus −1.96 (23.79%)***
	WIS	516 versus 719 (28.23%)***	516 versus 588 (12.29%)
5–13 weeks ahead	accuracy	0.29 versus 0.26 (11.41%)***	0.29 versus 0.21 (37.86%)***
	log score	−2.2 versus −2.58 (46.44%)***	−2.2 versus −2.76 (75.8%)***
	WIS	797 versus 962 (17.08%)***	797 versus 956 (16.59%)***
14–26 weeks ahead	accuracy	0.27 versus 0.26 (4.52%)***	0.27 versus 0.16 (64.51%)***
	log score	−2.22 versus −2.57 (42.3%)***	−2.22 versus −2.89 (95.16%)***
	WIS	791 versus 947 (16.44%)***	791 versus 1005 (21.25%)***
peak week−three months	accuracy	0.62 versus 0.58 (7.7%)	0.62 versus 0.57 (9.3%)***
	log score	−3.78 versus −3.74 (-4.02%)***	−3.78 v −4.29 (66.46%)***
	WIS	6 versus 7 (13.45%)***	6 versus 9 (29.27%)***
peak week−six months	accuracy	0.33 versus 0.33 (0.4%)	0.33 versus 0.27 (19.8%)
	log score	−3.48 versus −3.68 (22%)***	−3.48 versus −4.33 (134.45%)***
	WIS	13 versus 15 (9.68%)***	13 versus 18 (24.23%)***
peak intensity−three months	accuracy	0.31 versus 0.4 (-22.2%)*	0.31 versus 0.27 (17.94%)
	log score	−1.65 versus −2.16 (66.64%)***	−1.65 versus −1.97 (37.13%)***
	WIS	1309 versus 1513 (13.48%)	1309 versus 1445 (9.38%)
peak intensity−six months	accuracy	0.25 versus 0.31 (−17.69%)	0.25 versus 0.25 (2.83%)
	log score	−1.14 versus −1.59 (56.23%) ***	−1.14 versus −1.33 (21.05%) ***
	WIS	1694 versus 1757 (3.58%)	1694 versus 1813 (6.58%)*
total−three months	accuracy	0.29 versus 0.27 (8.65%)***	0.29 versus 0.22 (31.87%)***
	log score	−1.4 versus −1.65 (28.52%)**	−1.4 versus −1.74 (40.48%)
	WIS	7878 versus 9215 (14.51%)	7878 versus 9405 (16.24%)***
total−six months	accuracy	0.34 versus 0.34 (−0.49%)	0.34 versus 0.31 (11.6%)
	log score	−2 versus −2.36 (43.28%)*	−2 versus −2.37 (44.22%)***
	WIS	12 797 versus 15 273 (16.21%)	12 797 versus 14 664 (12.73%)***

### 3.3. The best-performing forecast approach

Overall, the SIRS ($\tilde{AH/T}/Vary$)-EAKF approach (i.e. using the SIRS model with seasonal-trend-based climate modulation and mixed immunity assumptions) outperformed all other approaches (table 1 and figure 2). The SIRS ($\tilde{AH/T}/Vary$)-EAKF approach consistently ranked the highest (five out of six instances) or second highest (i.e. accuracy predicting the seasonal targets; table 1). This superior performance is also evident from figure 2 for individual forecast targets (see the turquoise-triangle-dotted lines above others). The SIRS ($\tilde{AH/T}/Vary$)-EAKF approach significantly improved the overall point prediction accuracy by 3% and 24%, log score by 39% and 64%, and WIS by 18% and 19%, compared with the benchmark SARIMA approach and baseline SIRS (null)-EAKF approach, respectively (table 2).

**Figure 4.**
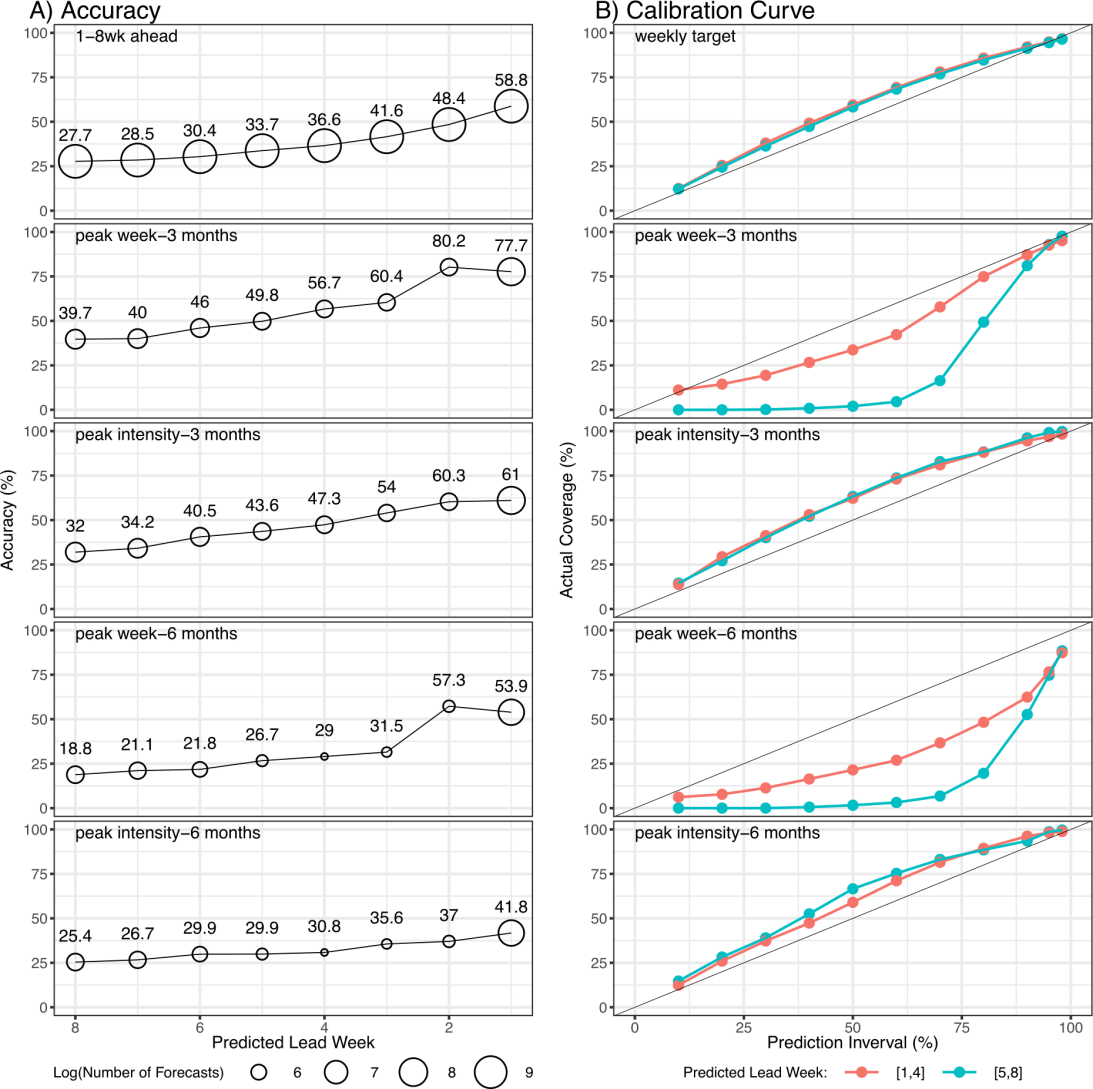
Performance of the best-performing approach by target and predicted lead time. Left panel shows forecast accuracy aggregated by lead time (see *x*-axis) for each target (see subpanel titles); dot sizes indicate number of forecasts falling in the strata (see legend) and numbers show the accuracies. Right panel shows calibration curves for the corresponding targets (see subpanel titles), further segregated into two categories per lead time (red = 1–4 weeks leads; green = 5–8 weeks leads). For peak week and peak intensity, the predicted lead time is computed as the difference between the predicted peak week and the week of forecast initiation (e.g. a forecast predicting a peak in five weeks has a lead time of five weeks).

We further examine forecast performance of the SIRS ($\tilde{AH/T}/Vary$)-EAKF approach, over different predicted lead times. For all forecast targets, the accuracy increased substantially as the lead time shortened (figure 4). The one and two weeks ahead predictions were accurate (i.e. within 25% of observed ILI+) approximately half of the time (first row in figure 4*a*). This approach was also able to accurately predict the peak week (within ±1 week of the observation) over the next three months up to approximately four weeks in advance (accuracy ranged from 78% for one week lead to 57% for four weeks lead; figure 4*a*, second row). The accuracy predicting peak intensity over the next three months was also relatively accurate up to approximately three weeks in advance (54–61% accuracy; figure 4*a*, third row). Considering that influenza epidemics in temperate regions typically last for 8–20 weeks (mean approx. three months), these accuracies predicting the peak timing and intensity over a three months horizon are comparable to those reported for US cities (versus fig. 1 of [10]) and combined measures for multiple temperate regions (versus fig. 1*a* of [11]). For the six months seasonal targets (peak week and peak intensity; figure 4*a*, last two rows), the forecast accuracies were substantially lower (less than 50% accurate for all lead times for peak intensity); however, peak week forecasts were greater than 50% accurate for short lead times 1–2 weeks relative to the predicted peak over a six months horizon.

As noted in §2, the reliability of forecasts (i.e. the forecast probability should be approximately the same as the observed event frequency) is an important forecast quality. Thus, we further examine the forecast reliability by forecast targets and predicted lead time. In general, the calibration curve of the incidence forecasts (1–8 weeks ahead forecasts in figure 4*b*, first row; peak intensity in figure 4*b*, third row for three months horizon and sixth row for six months horizon) aligned with the 1 : 1 line, indicating well-calibrated forecast probability for these targets. However, for the peak timing forecasts (figure 4*b*, second row for three months horizon and fourth row for six months horizon), the calibration curves tended to curve downward below the 1 : 1 line, indicating those forecasts were under-dispersed.

## 4. Discussion

Previous studies have found it challenging to predict influenza epidemics in the (sub)tropics, owing to much more irregular influenza activities in those regions [11,14,42]. Here, we demonstrate that incorporating mechanisms of climate modulation on influenza transmission can substantially improve forecast performance in subtropical Hong Kong, over 21 years including the 2009 A(H1N1)pdm09 pandemic. We show the forecast accuracy of our best-performing approach is comparable to that reported for temperate regions including the USA [10,11]. Importantly, here we have not only examined forecast performance for short-term targets (one to four weeks ahead predictions) but also mid-/long-range targets (up to three and six months ahead, respectively) and peak targets. Mid-/long-range targets and peak targets are more challenging to forecast, especially for the (sub)tropics, where influenza epidemics are more diverse; yet these forecasts can support more proactive public health planning, particularly on the longer timescales (up to six months) examined here. The improvements developed in this study, including for mid-/long-range and peak targets thus showcase the promise of accurate influenza forecasts in the (sub)tropics.

A major challenge for accurate infectious disease forecasting is nonlinear transmission dynamics. This nonlinearity implies potential exponential error growth over time and consequently chaotic dynamics if the system is not well-constrained. To address this challenge, here we harness a strong driver of influenza transmission dynamics—the seasonal risk of infection (i.e. seasonality) due to climate modulation—and incorporate mechanistic climate-forced seasonality models in our forecast approaches. The extensive testing in this study demonstrates that the climate-forced seasonality models enable more accurate modelling of influenza epidemics and superior forecast accuracy. As noted in §1, most current infectious disease forecasts are made for the near future with one to four weeks lead (i.e. short-term forecasts; e.g. in [3,43]), as accuracy degrades over longer horizon. Compared with the SARIMA benchmark approach, our best-performing climate-forced SIRS-EAKF approach showed superior forecast skills (improved accuracy, log score and WIS) for short-term forecasts, as well as mid-range (e.g. 5–13 weeks ahead, i.e. up to three months) and long-range (e.g. 14–26 weeks ahead, i.e. up to six months) forecasts (figure 2 and table 2). Consistently, we have also found improved COVID-19 forecasts using climate-forced seasonality models for several states in the USA across diverse climate conditions [37]. Together, these study findings highlight seasonality as a key transmission driver for respiratory viruses and the importance of modelling this mechanism to improve forecasts of these diseases.

Both climate modulations tested here model the impact of humidity [15,16] and temperature [7,21] on influenza virus survival and transmission, based on laboratory findings. The first climate modulation (AH/T) [20] uses climate data to directly model such impacts, whereas the second one ($\tilde{AH/T}$) uses a seasonal trend per the AH/T model estimates (see §2). Interestingly, the more flexible AH/T model did not outperform the seasonal-trend-based $\tilde{AH/T}$ model, particularly in predicting long-range targets (table 1, better rankings of $\tilde{AH/T}$ models for seasonal targets, and figure 2, where the turquoise lines are above the blue lines). Inspection of the forecasts suggests the inferior performance of AH/T modulation may be due to its higher flexibility leading to more volatile forecasts (e.g. figure 1 columns 2 and 3, wider forecast ensemble spread for lead times greater than 10 weeks in rows 3 and 4 using AH/T versus rows 5 and 6 using $\tilde{AH/T}$). This comparison again highlights the challenges facing long-range infectious disease forecasts. More importantly, it also suggests seasonal-trend-based climate modulations, with less flexibility and thus stronger constraints, may be advantageous for long-range forecasts.

Our best-performing forecast approach also included a mixed immunity model developed in our prior work [20] to more flexibly account for the waning of prior immunity, including cross-immunity, which has been shown to help shape influenza evolutionary and epidemic dynamics [44]. Compared with SIRS models with the classic exponential immune waning formulation, this mixed immunity formulation also helped improve forecast accuracy (figure 3*c*), albeit not as substantial as the climate modulations. Given its simplicity and superior forecast performance, this immunity-waning formulation (equation (2.3)) provides a simple alternative to help model the epidemiological impact of the interactions among cocirculating influenza viruses and can be readily incorporated in operational forecasts to help predict the resulting epidemic dynamics.

Another strength of this study is the extensive effort for forecast calibration. We tested a large combination of settings related to the spread of model ensemble during both the training and forecast stages (see §2). We were able to calibrate the incidence forecasts (*n*-week ahead and peak intensity forecasts; figure 4*b* for one to eight weeks leads and electronic supplementary material figure S2 for all forecasts combined). However, calibration of peak timing (figure 4*b* and electronic supplementary material, figure S2) proved more challenging. We suspect this may be in part due to the diverse epidemic dynamics in the (sub)tropics—e.g. as shown in figure 1, there could be 0, 1 or 2 epidemics in a span of six months. As such, the probability distribution of peak week may not be unimodal, rendering it more challenging to calibrate. Accordingly, improving the forecast system’s ability to predict the emergence of new influenza strains and/or antigenic innovations—a key driver of multi-modal epidemic waves—could help better capture the corresponding multi-modality of peak week distribution and help improve the forecast calibration. We plan to explore this strategy in future work.

This study is limited in that we only tested the forecast approaches for Hong Kong, given data availability. The likely better data quality for Hong Kong may contribute to the better forecast performance reported here. Future work can extend to test approaches developed here for other (sub)tropical regions. As a first step to examine forecast improvement with the use of climate conditions, we opted for simpler models focusing on including climate modulation and sidestepped several other key factors for influenza transmission. These include antigen innovations, cocirculation of influenza strains and cross-immunity, heterogeneous population mixing (e.g. for different age groups) and vaccination. Nonetheless, by combining multiple technical innovations in infectious disease forecasting, this study demonstrates accurate influenza forecasts are possible for the (sub)tropics. Future efforts extending the climate-forced epidemic models to include the factors noted above may additionally improve forecast performance in the (sub)tropics.

## Data Availability

Data and model code are publicly available at Zenodo: [45] Supplementary material is available online [46].
